# Validation of prediction algorithm for risk estimation of intracranial aneurysm development using real-world data

**DOI:** 10.1038/s41598-023-41986-6

**Published:** 2023-09-05

**Authors:** Tackeun Kim, Jisu Choi, Won-Ju Park, Seunghyeon Cho, Yeongjae Yoo, Hyeonjun Kim, Juhee Cho, Jin-Deok Joo, Chang Wan Oh

**Affiliations:** 1 TALOS Corp, 160, Yeoksam-ro, Gangnam-gu, Seoul, 06249 Republic of Korea; 2 Department of Neurosurgery, Seoul National University Bundang Hospital, 82, Gumi-ro 173 Beon-gil, Bundang-gu, Seongnam-si, Gyeonggi-do 13620 Republic of Korea; 3 Department of Neurosurgery, Seoul National University College of Medicine, 101 Daehak-ro, Jongno-gu, Seoul, 03080 Republic of Korea; 4 Department of Occupational and Environmental Medicine, Chonnam National University Medical School, Chonnam National University Hwasun Hospital, 322 Seoyang-ro, Hwasun, 58128 Republic of Korea; 5 Department of Clinical Research Design and Evaluation, Samsung Advanced Institute for Health Sciences and Technology, 115, Irwon-ro, Gangnam-gu, Seoul, 06355 Republic of Korea; 6 Department of Neurosurgery, Jeju National University Hospital, 15, Aran 13-gil, Jeju-si, Jeju-do 63241 Republic of Korea

**Keywords:** Predictive markers, Cerebrovascular disorders, Stroke, Risk factors

## Abstract

Intracranial aneurysm (IA) is difficult to detect, and most patients remain undiagnosed, as screening tests have potential risks and high costs. Thus, it is important to develop risk assessment system for efficient and safe screening strategy. Through previously published research, we have developed a prediction model for the incidence risk of IA using cohort observational data. This study was designed to verify whether such a prediction model also demonstrates sufficient clinical performance in predicting the prevalence risk at the point of health screening, using cross-sectional data. The study population comprised individuals who visited the Chonnam National University Hwasun Hospital Health Promotion Center in Korea for voluntary medical checkups between 2007 and 2019. All participants had no history of cerebrovascular disease and underwent brain CTA for screening purpose. Presence of IA was evaluated by two specialized radiologists. The risk score was calculated using the previously developed AI model, and 0 point represents the lowest risk and 100 point represents the highest risk. To compare the prevalence according to the risk, age-sex standardization using national database was performed. A study collected data from 5942 health examinations, including brain CTA data, with participants ranging from 20 to 87 years old and a mean age of 52 years. The age-sex standardized prevalence of IA was 3.20%. The prevalence in each risk group was 0.18% (lowest risk, 0–19), 2.12% (lower risk, 20–39), 2.37% (mid-risk, 40–59), 4.00% (higher risk, 60–79), and 6.44% (highest risk, 80–100). The odds ratio between the lowest and highest risk groups was 38.50. The adjusted proportions of IA patients in the higher and highest risk groups were 26.7% and 44.5%, respectively. The median risk scores among IA patients and normal participants were 74 and 54, respectively. The optimal cut-off risk score was 60.5 with an area under the curve of 0.70. We have confirmed that the incidence risk prediction model built through machine learning also shows viable clinical performance in predicting prevalence risk. By utilizing this prediction system, we can effectively predict not only the incidence risk but also the prevalence risk, which is the probability of already having the disease, using health screening data. This may enable us to consider strategies for the early detection of intracranial aneurysms.

## Introduction

Intracranial aneurysm (IA) is among the most dangerous diseases owing its clinical course related to subarachnoid hemorrhage (SAH), which is led by rupture of it^1^. The case fatality of SAH is known to be high as 35%, and only a third of survivors can resume their premorbid work^2^. To prevent SAH, adequate treatment should be provided for the patients with rupture prone unruptured IA. However, detecting unruptured IA mainly relies on the incidental findings because there are few symptoms related to IA. Besides, the guidelines for IA screening are covering very narrow extent of populations such as those with familial history (≥ two family members with IA or SAH) or genetic diseases (autosomal dominant polycystic kidney disease, microcephalic osteodysplastic primordial dwarfism)^3,4^. Moreover, diagnostic modalities, such as computed tomography angiography (CTA) or magnetic resonance angiography (MRA), cannot be easily performed due to the potential risk of side effects such as radiation and contrast agents or very high cost. Thus, the prevalence and incidence of IA tends to be estimated very low. Indeed, only 0.44% of populations during a 8.8 million person-year observation were diagnosed as having unruptured IA or SAH^1^. Contrarily, the prevalences among participants who underwent MRA were reported as 2.8–7.0%^5–7^. To put it all together, most IA patients remain undiagnosed because they have never undergone adequate examinations.

As described above, screening tests, such as CTA or MRA, cannot be recommended for all populations due to potential risks (medical supply capacity, radiation exposure, contrast agent side effects, claustrophobia) and high cost. Thus, we need to identify the individual risk for IA and find high-risk populations requiring screening tests. In our previously published research, we constructed a prediction model that is aimed at determining the incidence risk of IA. This was achieved by employing cohort observational data, which allowed us to analyze the likelihood of IA developing over time. In the current study, we aimed to verify whether this predictive model can also demonstrate sufficient clinical performance when applied to predicting the prevalence risk of IA at the time of health screening using cross-sectional data.

## Materials and methods

### Developed AI model

We implemented machine learning to predict the risk of developing IA, based on previous study. Our data was derived from the Korean national sample cohort, which was created by randomly selecting 2% of the population stratified from the entire population data. We extracted data for subjects who had undergone general health examinations from 2009 to 2013 from this cohort.

Out of the eligible 427,362 subjects, 1067 (0.25%) were identified as having intracranial aneurysms (IA), as indicated by diagnostic codes I671or I60x, and the remaining 426,295 subjects were allocated to the control group. After excluding 93 patients who had not undergone computed tomography (CT), magnetic resonance (MR), or cerebral angiography correlating with the diagnostic codes, 974 (0.23%) subjects were finally allocated to the IA group.

The health examination data included 21 variables: age, sex, body mass index, waist circumference, systolic/diastolic blood pressures, fasting glucose, total cholesterol, high-density lipoprotein, low-density lipoprotein, triglyceride, hemoglobin, creatinine, gamma-glutamyl transferase, aspartate aminotransferase, alanine aminotransferase, smoking status (never, ex-, or current smoker), and familial histories of stroke, hypertension, heart disease, and diabetes.

For model training and evaluation, we divided all subjects into training (70%) and test (30%) datasets via random allocation. The training dataset consisted of 299,088 subjects, including 682 (0.23%) IA cases, and the test dataset included 128,181 subjects, with 292 (0.23%) IA cases.

**Figure 1:**
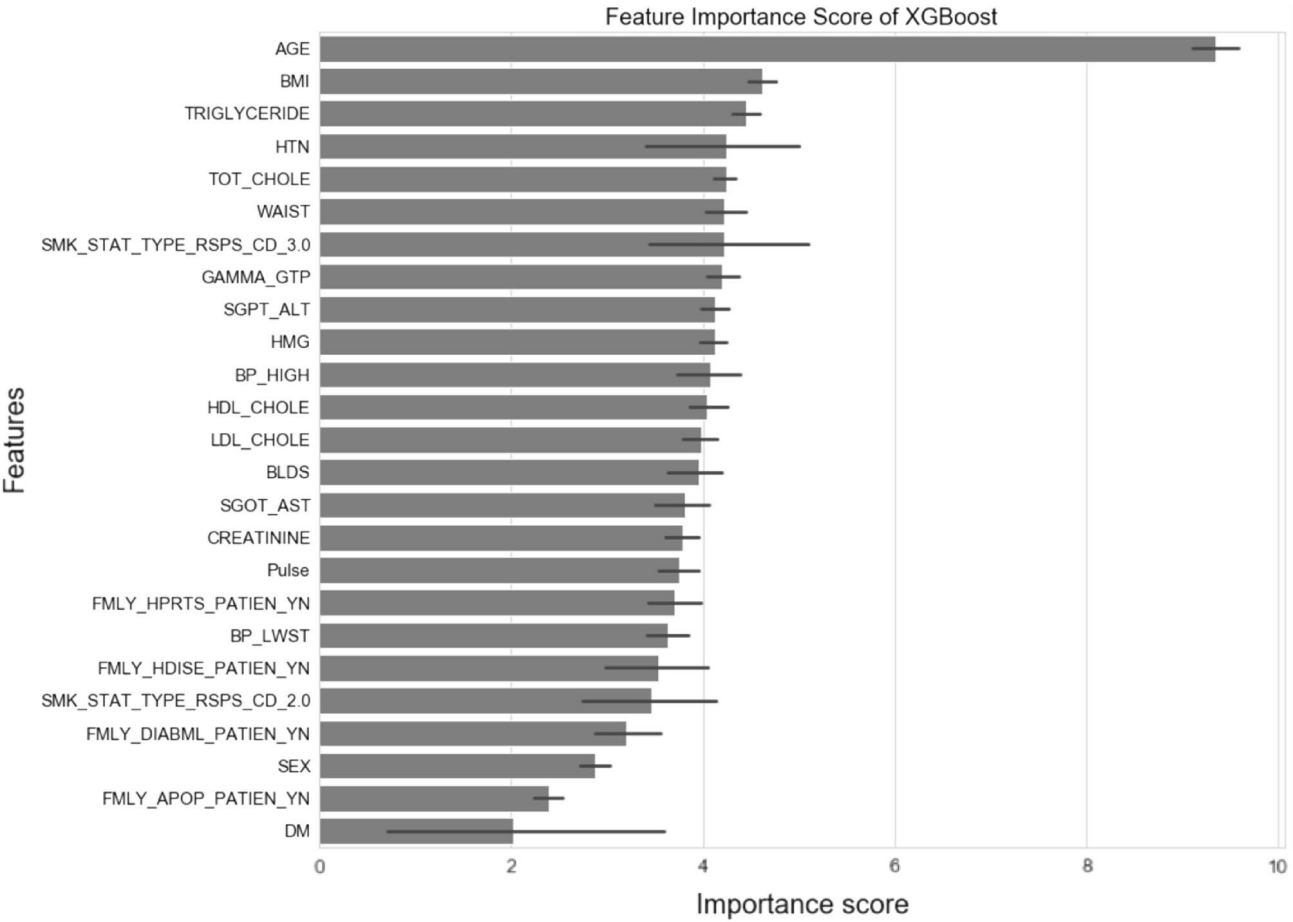
Feature importance scores of the model. The length of the bar represents the score, and the length of the line indicates the 95% confidence interval. *BMI* body mass index, *HTN* hypertension, *TOT_CHOLE* total cholesterol, *WAIST* waist circumference, *SMK_STAT_3.0/2.0* current/ex smoker, *HMG* hemoglobin, *BP_HIGH/BP_LWST* systolic/diastolic blood pressures, *BLDS* fasting glucose, *Pulse* pulse pressure, *FMLY_HPRTS/HDISE/DIABML/APOP* familial history of hypertension/heart disease/diabetes/stroke, *DM* diabetes.

Throughout our experiments, the XGBoost model, trained with hyperparameters that included a learning rate of 0.1, maximum depth of 4, minimum child weight of 4, and subsampling rate of 0.8, exhibited the best performance. This performance was measured by the area under the receiver operating characteristic curve (AUROC), with the highest AUROC being 0.765 for the test dataset^3^. The feature importances of the variables applied to the model are summarized in Fig. 1.

### Study populations

The study participants for validating our model were those who visited the Chonnam National University Hwasun Hospital (CNUHH) Health Promotion Center of the Republic of Korea for voluntary medical checkups between January 2007 and December 2019. We conducted a retrospective review of the participants’ medical records. The participants’ data were anonymized and stored in the CNUHH Clinical Data Warehouse. The data were used for research purposes in accordance with the Personal Information Protection Act of the Republic of Korea. Each participant provided electronic informed consent regarding the collection and use of personal information before the checkup was conducted. All participants in this study have no history of cerebrovascular disease and are currently asymptomatic, as they had a consultation in the treatment department instead of a health checkup if they had any special symptoms. The study protocol was approved by the Institutional Review Board (IRB) of CNUHH (IRB number CNUHH-2022-207) and all of research processes were performed in accordance with relevant guidelines and regulations.

### Data collection

The participants were interviewed by trained physicians using a structured questionnaire with items on smoking and familial history of diseases (stroke, diabetes mellitus, heart disease, hypertension, and so on). Smokers were defined as those who smoked ≥ 100 cigarettes in their lifetime. A person who had quit smoking for ≥ 1 year was defined as a former smoker. Height and weight were measured in the upright position without shoes and socks. Waist circumference was measured at the mid-point between the 12th rib and anterior superior iliac spine. The body mass index (BMI) was calculated by dividing the weight (kg) by the squared height (m^2^). Blood pressure was measured in the left upper arm using a regularly calibrated electronic sphygmomanometer, after the participants has rested for > 10 min. Blood tests were performed after fasting for > 12 h. Brain CTA was performed using a 128-slice dual source CT scanner (SOMATOM Definition Flash, Siemens, Germany) after 2011. Prior to 2011, a 64-slice CT scanner (LightSpeed VCT, GE Healthcare, Milwaukee, WI, USA) was used. After imaging, two experienced radiology specialists cross-examined the presence of IA.

### Inference with trained algorithm

Twenty-one variables (age, sex, BMI, waist circumference, systolic and diastolic blood pressures, fasting glucose, total cholesterol, high-density lipoprotein, low-density lipoprotein, triglyceride, hemoglobin, creatinine, gamma-glutamyl transferase, aspartate aminotransferase, alanine aminotransferase, smoking status, and familial histories of stroke, hypertension, heart disease, and diabetes mellitus) were substituted to the inference algorithm. The result values returned by the inference algorithm were risk scores that were presented as integers from 0 to 100, with higher scores indicating a higher risk).

### Statistical analyses

We used Python (version 3.11; Python software foundation; https://www.python.org) and R (version 4.2.0; the R Foundation for Statistical Computing; https://www.r-project.org) for inference and statistical analyses.

Age-sex standardization was performed using a reference dataset that had been prepared as a test dataset in the previous research^4^. It comprises 128,181 individuals with general health examination data who were labeled for the presence of IA. As it was curated by stratified random selection among the whole national population, it can represent the demographic characteristics of the entire population who participated in the healthcare screening program^8^. Age was grouped into 10-year-old units for standardization. Using age group and sex, the propensity scores were calculated for each individual. Based on the propensity scores, the weights were determined and applied to produce age-sex standardized prevalence of intracranial aneurysm. The risk score was defined as a ranking score calculated from probability, where 0 is the lowest and 100 is the highest.

Generally, *p*-value < 0.05 was accepted for statistical significance. Continuous variables were presented as mean ± standard deviation and analyzed by using Student t-test. As the risk score was ranked, it was presented as median (interquartile range, IQR) and analyzed by using Mann–Whitney U test. Categorical variables were presented as proportion (%) and analyzed by using chi square test or Fisher’s exact test according to the expected counts. To evaluate the linear trend of the risk score, risk scores were divided into the following quintile groups as defined in previous work^4^: 0–19, lowest risk; 20–39, lower risk; 40–59; mid risk; 60–79, higher risk; and 80–100, highest risk. The extended Mantel Haenszel chi square test was used to calculate statistical significance of the scoring system.

## Results

Through a data collection process, a total of 5942 health examination data, including brain CTA data, were collected. The participants’ age range of was 20 to 87 years, with a mean of 52.15 ± 8.82 years. The proportion of female participants was 33.6%. Among them, 237 were diagnosed with IA. Thus, the crude prevalence of IA was 4.00% (95% confidence interval [CI] 3.52–4.53). The other variables of the enrolled participants are summarized in Table 1.

**Table 1:** Summarization of data.

Factor	NHIS	CNUHH	*p* value	CNUHH	*p* value
	n = 128,181	n = 5942		age/sex adjusted	
Age group					
20 ≤ , < 30	14.3%	0.3%	< 0.001	14.3%	1.000
30 ≤ , < 40	17.3%	6.5%		17.3%	
40 ≤ , < 50	28.5%	32.8%		28.5%	
50 ≤ , < 60	21.9%	41%		21.9%	
60 ≤ , < 70	11.8%	15.9%		11.8%	
70 ≤ , < 80	5.4%	3.2%		5.4%	
80 ≤	0.9%	0.2%		0.9%	
Sex (female)	51.2%	33.6%	< 0.001	51.2%	1.000
BMI (kg/m^2^)	23.55 ± 3.29	24.48 ± 2.86	< 0.001	24.10 ± 3.57	< 0.001
Waist circumference (cm)	79.41 ± 9.31	83.20 ± 8.37	< 0.001	81.45 ± 10.16	< 0.001
Systolic BP (mmHg)	121.18 ± 14.68	121.06 ± 15.80	0.543	118.14 ± 16.46	< 0.001
Diastolic BP (mmHg)	75.56 ± 9.93	71.67 ± 10.57	< 0.001	69.75 ± 10.69	< 0.001
Glucose (mg/dl)	95.07 ± 16.12	103.34 ± 25.53	< 0.001	99.61 ± 23.55	< 0.001
Total cholesterol (mg/dl)	193.67 ± 35.88	194.67 ± 37.17	0.069	192.81 ± 36.47	< 0.001
LDL cholesterol (mg/dl)	113.24 ± 32.85	122.81 ± 34.17	< 0.001	120.99 ± 33.84	< 0.001
HDL cholesterol (mg/dl)	55.85 ± 13.81	49.29 ± 11.77	< 0.001	51.00 ± 12.02	< 0.001
Triglyceride (mg/dl)	122.28 ± 72.61	128.42 ± 93.19	< 0.001	123.17 ± 94.31	0.013
Hemoglobin (g/dl)	13.86 ± 1.61	14.67 ± 1.51	< 0.001	14.35 ± 1.52	< 0.001
Creatinine (mg/dl)	0.89 ± 0.22	0.95 ± 0.18	< 0.001	0.90 ± 0.18	< 0.001
AST (IU/L)	23.74 ± 8.72	28.30 ± 16.07	< 0.001	28.65 ± 21.02	< 0.001
ALT (IU/L)	22.97 ± 14.11	28.98 ± 19.39	< 0.001	30.16 ± 28.11	< 0.001
GGT (IU/L)	31.31 ± 28.85	44.76 ± 49.78	< 0.001	37.25 ± 44.07	< 0.001
Stroke	5.5%	8.3%	< 0.001	7.1%	< 0.001
DM	9.2%	16.1%	< 0.001	15.3%	< 0.001
Family history					
Heart disease	3.4%	4.6%	< 0.001	4.6%	< 0.001
Hypertension	11.7%	24.4%	< 0.001	24.5%	< 0.001
Smoking					
Never	62.7%	50.4%	< 0.001	65.3%	< 0.001
Former	12.7%	26.5%		15.9%	
Current	24.6%	23.1%		18.8%	

Continuous variables are presented as mean ± standard deviation. Categorical variables are represented as percentages.

*NHIS* National Health Insurance Service, *CNUHH* Chonnam National University Hwasun Hospital, *BMI* body mass index, *BP* blood pressure, *LDL* low-density lipoprotein, *HDL* high-density lipoprotein, *AST* aspartate aminotransferase, *ALT* alanine transaminase, *GGT* gamma-glutamyl transferase, *DM* diabetes mellitus.

**Figure 2:**
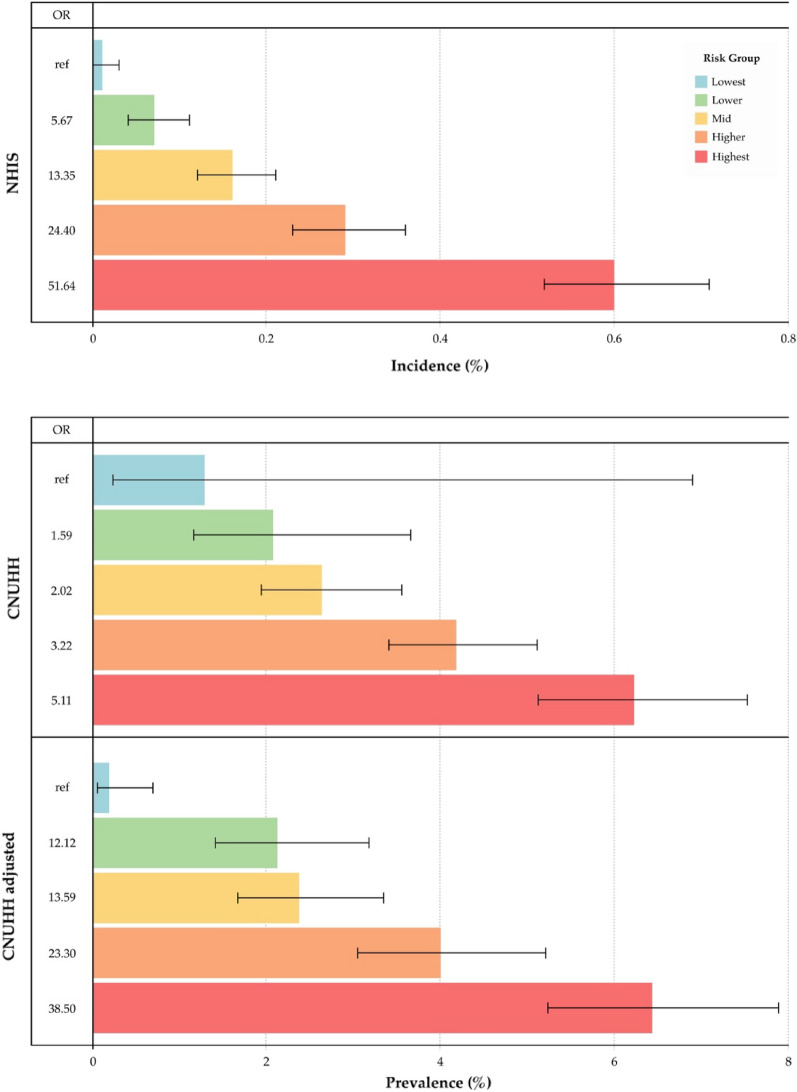
Incidence and prevalence by risk groups. The upper bar chart shows the incidence of IA by pentile risk groups as described in a previous study using the National Health Insurance Service (NHIS) dataset. Middle and lower bar charts present the prevalence of IA by pentile risk groups inferred by the algorithm using crude and adjusted Chonnam National University Hwasun Hospital (CNUHH) datasets, respectively. Each error-bar represents 95% confidence interval. *OR* odds ratio.

Among 78 participants who were classified as the lowest risk group, one participant was diagnosed with IA (crude prevalence, 1.28%, 95% CI 0.23–6.91). The crude prevalence (95% CI) of the lower, mid, higher, and highest risk groups was 2.03% (1.14–3.59), 2.56% (1.89–3.47), 4.01% (3.27–4.91), and 6.23% (5.13–7.54), respectively. The odds ratios of each group compared with the lowest risk group were 1.59, 2.02, 3.22, and 5.11, respectively (Figure 2 and Table 2). Among all IA cases, 40.5% (96 out of 237) and 37.6% (89 out of 237) occurred in the highest and higher risk groups, respectively. The proportion of IA patients in the higher and highest risk groups reached 78.1%.

**Table 2:** Incidences, prevalences and odds ratios by predicted risk groups.

Dataset	Predicted risk group	No. of subjects	No. of IADiagnosed	Incidence (95% CI)	Prevalence (95% CI)	Odds ratio (95% CI)
NHIS	Lowest	25,636	3	0.01 (0.00–0.03)		ref
	Lower	25,636	17	0.07 (0.04–0.11)		5.67 (1.66–19.35)
	Mid	25,636	40	0.16 (0.12–0.21)		13.35 (3.97–53.96)
	Higher	25,636	73	0.29 (0.23–0.36)		24.40 (7.69–77.43)
	Highest	25,637	154	0.60 (0.52–0.71)		51.64 (16.47–161.9)
	Sum	128,181	287	0.22 (0.20–0.25)		
CNUHH	Lowest	78	1		1.28 (0.23–6.91)	ref
	Lower	543	11		2.03 (1.14–3.59)	1.59 (0.20–12.50)
	Mid	1562	40		2.56 (1.89–3.47)	2.02 (0.28–14.92)
	Higher	2217	89		4.01 (3.27–4.91)	3.22 (0.44–23.42)
	Highest	1542	96		6.23 (5.13–7.54)	5.11 (0.70–37.15)
	Sum	5942	237		4.00 (3.52–4.53)	
CNUHH adjusted	Lowest	1008.5	1.8		0.18 (0.05–0.69)	ref
	Lower	1056.0	22.4		2.12 (1.41–3.18)	12.12 (2.65–55.46)
	Mid	1293.8	30.7		2.37 (1.67–3.35)	13.59 (3.02–61.25)
	Higher	1269.9	50.8		4.00 (3.05–5.22)	23.30 (5.26–103.3)
	Highest	1313.7	84.6		6.44 (5.24–7.90)	38.50 (8.78–169.0)
	Sum	5942.0	190.2		3.20 (2.78–3.68)	

*CI* confidence interval, *NHIS* National Health Insurance Service dataset, *CNUHH* Chonnam National University Hwasun Hospital, *ref* reference.

**Figure 3:**
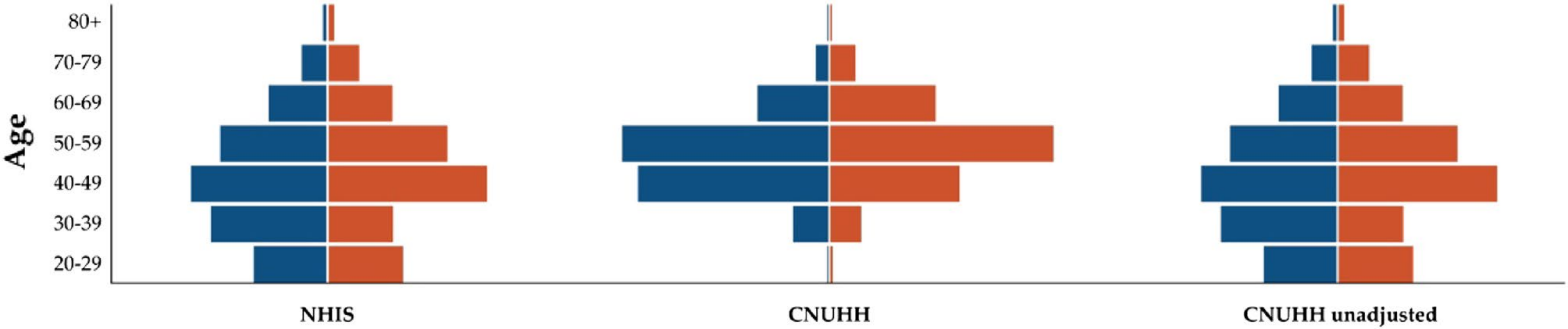
Butterfly chart of the population structures. Blue represents the proportion of males, while orange signifies the proportion of females. Left, population structure of the National Health Insurance Service (NHIS) dataset, which represents the national distribution of individuals who underwent healthcare screening. Middle, population structure of the crude Chonnam National University Hwasun Hospital (CNUHH) dataset. Right, population structure of age/sex adjusted for the CNUHH dataset.

As the distributions of age and sex were significantly different from the national population structure, age-sex standardization was performed as described above. As a result, the distributions of age and sex were adjusted to fit the reference dataset (National Health Insurance Service [NHIS]); the age-sex distribution of each dataset is summarized in Table 1 and Figure 3. The age-sex standardized prevalence of IA was 3.20% (95% CI 2.78–3.68) among the Chonnam National University Hwasun Hospital (CNUHH) dataset. Applying each weight for age-sex standardization, the prevalence (95% CI) of the lowest, lower, mid, higher, and highest risk groups was 0.18% (0.05–0.69), 2.12% (1.41–3.18), 2.37% (1.67–3.35), 4.00% (3.05–5.22), and 6.44% (5.24–7.90), respectively. (Table 2) The odds ratio between the lowest and highest risk groups was 38.50 (8.78–169.0). As to the linear trend of the predicted score, the extended Mantel Haenszel Chi Square value was calculated as 78.88, and the *p*-value was < 0.001. The adjusted proportions of IA patients in the higher and highest risk groups were 26.7% and 44.5%, respectively. The median risk scores among the IA patients and normal participants were 74 (IQR 56–90) and 54 (IQR 29–76), respectively (*p* < 0.001). The optimal cut-off risk score was 60.5 with 0.70 (95% CI 0.69–0.70) of the area under the curve.

## Discussion

Annual medical expenditures covered by the NHIS for SAH was 120 billion Korean Won (KRW) in 2010, which had gradually increased by an annual growth rate of 7.74%, reaching 272 billion KRW in 2021^9^. At the exchange rate of December 2022, it is approximately 215 million USD. Even so, this cost is limited to what is covered by the insurance, and the amount of non-insured treatment is not included. Moreover, SAH results in death and severe disability in > 50% of all patients. Considering not only the loss of the patient's labor force, but also the family's labor force sacrificed for the patient's care, the socio-economic loss due to SAH becomes even greater. Considering the high cost of SAH, there have been few researches regarding the epidemiology of IA. Although a few studies reported the prevalence of IAs as 2.8–7.0%, the study populations were limited to single institutes or provinces^5–7^. This is because, in most countries, there are no databases on the state of medical use at the national level. Using the national database of the Republic of Korea, the incidence and risk factors of IA were reported^1^. The estimated age-sex standardized incidence of IA was 52.2 per 100,000 person-year.

Unlike other critical diseases that are costly, SAH can be prevented if we can find IAs before rupture, because most spontaneous cases of SAH are caused by rupture of IA. However, considering together the estimated incidence and prevalence calculated from the participants who had undergone neurovascular imaging, most IA patients have been undiagnosed because they did not undergo appropriate examinations^1,5–7^. In fact, from 2008 to 2016, the number of national incident cases of unruptured IA had increased by 336.7%. The annual growth rate approached to 30%. Meanwhile, in the same time window, the number of SAH patients did not decrease, but rather increased by 2.4%^10^. The rates of detection and treatment for unruptured IA seemed to be the tip of the iceberg to reduce the number of SAH. This also supports the idea that most IA patients are undiagnosed.

The guidelines for screening IA are as follows: (1) patients with two family members or more with IA or SAH should be offered aneurysmal screening by CTA or MRA. The risk factors that predict a particularly high risk of aneurysm occurrence in such families include history of hypertension, smoking, and female sex; and (2) patients with a history of autosomal dominant polycystic kidney disease (ADPKD), particularly those with a familial history of IA, should be offered to undergo screening by CTA or MRA, and it is reasonable to offer CTA or MRA to patients with coarctation of the aorta or microcephalic osteodysplastic primordial dwarfism^3^. According to the familial history of IA, IA detection among first-degree relatives of those with sporadic SAH was approximately 4% (95% CI 2.6–5.8), with a somewhat higher risk among siblings than among children of those affected^11,12^. According to a meta-analysis of unruptured IA, the adjusted prevalence ratio was 3.4 (95% CI 1.9–5.9)^13^. However, considering the nationwide proportion of IA, populations with a familial history of IA are rare^10^. Similarly, the reason ADPKD was included in the screening guideline is that it shows a high prevalence rate as compared to the general population. Among ADPKD patients, the prevalence of IA was estimated to be up to 13.4%^14^. A previous meta-analysis reported that the prevalence ratio of ADPKD was 6.9 (95% CI 3.5–14.0)^13^. However, the estimated prevalence of ADPKD, which is the most common hereditary kidney disease in Korea, was approximately 1/10,000^15^. Moreover, only 0.3% of patients with unruptured IA had a history of polycystic kidney disease^16^. Therefore, this criterion can cover only a few number of the population. Moreover, the abovementioned meta-analysis included only approximately 600 patients with unruptured IA for the adjusted analysis.

Thus, a strategy to identify participants at a high risk of developing IA among the general populations is needed, because neurovascular imaging is very expensive to carry out for the whole population, and there is always a risk of potential side effects due to the use of contrast agent. In a previous work, we proposed a risk assessment algorithm for IA using a machine learning method that utilized national healthcare examination data^4^. It has shown good results from the perspective of incidence rate through cohort data using follow-up observational studies. However, to estimate the prevalence, which is the probability that intracranial aneurysms have already occurred at the time of health screening, we have determined that additional validation is needed which should utilize cross-sectional study data, including not only health screening results but also cerebrovascular imaging findings.

In this study, we validated the predictive power for the actual prevalence of intracranial aneurysms as confirmed by CTA, using the risk derived by applying the health screening results of 5942 individuals to the constructed artificial intelligence model. The standardized prevalence of IA was 6.44% (95% CI 5.24–7.90) in the highest risk group, which showed an odds ratio of 38.5 compared with 0.18% (95% CI 0.05–0.69) in the lowest risk group. Moreover, the dose–response relation was also found to be statistically significant. The standardized odds ratio of the highest risk group over the entire participants was 3.0 (95% CI, 2.2–4.0). Therefore, by adding the results of this study to previous research, the model predicting the risk of intracranial aneurysms can not only demonstrate acceptable performance in predicting future incidence but also ensure clinical utility in predicting the prevalence at the current point in time when receiving a health screening.

There are several limitations to our study. First, this study was designed as a retrospective validation; thus, we needed to consider potential biases. To overcome the population characteristics of those who visited CNUHH, a direct standardization was applied using data from a national database. As a result, age and sex distributions were statistically adjusted. Second, the diagnostic test performed was CTA. Although catheter angiography remains the gold standard for identifying IA, CTA shows comparable sensitivity and specificity along with MRA^17^. Moreover, considering the high procedure-related risk of catheter angiography, it is not suitable for use for screening purposes. In this study, two radiology specialists cross-read the CTA to enhance the diagnostic accuracy.

In conclusion, we have confirmed that the incidence risk prediction model built through machine learning also shows viable clinical performance in predicting prevalence risk. By utilizing this prediction system, we can effectively predict not only the incidence risk but also the prevalence risk, which is the probability of already having the disease, using health screening data. This may enable us to consider strategies for the early detection of intracranial aneurysms.

## Data Availability

The data are not available for public access because of patient privacy concerns but are available from the corresponding author on reasonable request approved by the institutional review boards of Chonnam National University Hwasun Hospital.
